# Which health care facilities do adult malawian antiretroviral therapy patients utilize during intercurrent illness? a cross sectional study

**DOI:** 10.1186/1472-6963-11-345

**Published:** 2011-12-21

**Authors:** Caroline Masangalawe, Akuzike Kandulu, Joep J van Oosterhout

**Affiliations:** 1 Department of Medicine, University of Malawi, College of Medicine, Blantyre, Malawi

**Keywords:** Health care utilization, antiretroviral therapy, Malawi, HIV

## Abstract

**Background:**

Antiretroviral therapy (ART) clinic populations have expanded enormously in the successful Malawi ART scale-up programme. Overcrowding, long waiting times and living far away from the clinic may affect the extent to which patients use their ART clinic for intercurrent illnesses.

**Methods:**

We interviewed patients of a large urban ART clinic in Blantyre, Malawi, during routine visits about the choice of health care facility during recent illness episodes.

**Results:**

Out of 346 enrolled adults, mean age 39.8 (range 18-70) years, 54.3% female, 202 (58%) reported one or more illness in the past 6 months, during which 85 (42.1%; 95%-confidence interval: 36.9-47.3%) did not utilize their own clinic. Long distance to the clinic was the main subjective reason, while low education attainment, rural residence, perceived mild illness and dissatisfaction with the ART service were associated with not using their own clinic in multivariate analyses. Of all participants, 83.6% were satisfied with the service provided; only 6.1% were aware of the full service package of the ART clinic.

**Conclusions:**

ART patients often seek health care outside their own clinic, which may have detrimental effects, and has consequences for ART counseling content and reporting of ART information in health passports.

## Background

Malawi has been seriously affected by the HIV pandemic. By 2010 there were more than 900,000 persons living with HIV/AIDS, of whom close to 225,000 were receiving antiretroviral therapy (ART) in a free national ART programme that has rapidly expanded since 2004 [1]. ART is offered at government, faith-based and private hospitals and health centers. According to the national ART guidelines [2], ART clinics should provide services that include management of opportunistic infections, malignancies and toxicities of ART as a minimum.

It is uncertain to what extent patients use their own ART clinic in case of intercurrent medical problems, such as HIV associated illnesses and side effects of ART. In the context of rapid scale up of ART, clinic populations have increased enormously and in combination with limited space and shortages of ART staff that are present in many clinics, aspects of the quality of ART services may be under pressure. As a result, patients might decide to use other facilities than their own ART clinic, anticipating less overcrowded circumstances, shorter waiting times and more privacy than experienced during previous ART visits. In the National ART programme, pre-ART counseling content may recommend patients to utilize closer-by health facilities for minor illnesses that are not ART toxicities.

Several studies have given insight into patterns of health care utilization in patients in the ART era in affluent countries, but these mainly focused on the quantity of health care consumption. In two American studies, the rate of emergency department admissions and hospitalizations was increased with not being on ART [3], having medical insurance, having high levels of pain, using illicit drugs, consuming alcohol and being female [4]. Studies from the USA and Canada showed that poor adherence to ART [5] and low CD4 counts and high viral loads [6] were associated with high health care utilization, while in Australia this was the case with ART patients with mental health problems [7]. It is unlikely that these results apply to circumstances in Malawi, where ART is provided with a public health approach under great human and financial resource strains. Few studies in sub-Saharan Africa have addressed health care utilization of ART patients. A retrospective cohort study of 212 South Africans found that health care utilization (defined as in- and outpatient hospital services, but excluding primary care visits) significantly decreased after the initiation of ART [8]. We could find only one study that specifically investigated the extent to which HIV patients looked for care outside their own clinic in case of illness and the reasons thereof. A study of 32 HIV infected persons from Johannesburg, South Africa found that many made simultaneous use of public, private and traditional health facilities, but after starting ART, patients more uniformly utilized their ART clinic as the primary source of health care [9].

Theoretically it can have several disadvantages if patients utilize other health care facilities instead of their own clinic in case of illness: the previous ART history or even the positive HIV status may not be communicated by the patient, detailed information concerning the treatment history will not be available, and smaller facilities may not be familiar with the less commonly used antiretroviral drug regimens that are prescribed in larger, central hospitals. If other health care facilities are utilized, it may also deprive the ART clinic of feedback about illness episodes of their patients, information that can provide important clues about (severe) toxicities, drug interactions and ART failure.

We therefore did a survey to evaluate the health care utilization of patients of the ART clinic of Queen Elizabeth Central Hospital (QECH), Blantyre, Malawi for intercurrent illness, and to find out the reasons for utilizing other health care facilities than their own clinic. We also assessed the patients' perception of the quality of care offered at their ART clinic. Such knowledge may help in improving the services that ART clinics offer.

## Methods

We conducted a cross sectional study at the ART clinic of Queen Elizabeth Central Hospital (QECH) in Blantyre, the largest government referral hospital in the southern region of Malawi. As of March 2010, more than 12,000 patients had been newly registered on ART in this clinic, and around 7,000 patients were retained on treatment [10]. Around 250 patients visit the clinic per day. We took a convenience sample, whereby the first 10 patients, who attended a morning or afternoon ART session, were aged 18 years or older, on ART for at least 6 months (irrespective of the regimen), and who had started treatment at the same clinic, were approached for enrolment during routine clinic attendance in June - July 2010. A structured questionnaire was employed for one-on-one interviews by investigators who were not part of the resident ART clinic staff. We collected demographic information, which included: age, gender, duration on ART, level of education, economic status and residence. A socio-economic score was obtained by determining how many of the following nine household items a participant possessed: electricity, paraffin lamp, radio, television, mobile telephone, bed with mattress, sofa set, table, chairs. Each participant therefore had a score ranging from 0 to 9. A similar socio-economic score based on household possessions was validated in sub-Saharan Africa [11]. The education level attained was determined by the number of years participants had successfully completed at school; repeated years were not counted. In Malawi primary schooling generally lasts 8 years, secondary schooling 4 to 6 years. We obtained information in relation to the choice of health care facility during illness, about patients' knowledge of services provided by the clinic and about their perceptions on the quality of care offered at the ART clinic.

We studied the utilization of health care facilities based on reported illness episodes during the 6 months prior to the current visit to the clinic. For uni-and multivariable analyses, we then grouped patients who reported an illness in the last six months into those who used QECH and those who used any other health care facility. When a patient reported multiple disease episodes, only the most recent one was included in the analysis. To assess whether the choice of health care facility was associated with sex, age, economic status, education level, urban/rural residence, travel time to the clinic, duration on ART, perceived severity of the illness, and satisfaction with the service of the own ART clinic, univariable logistic regression was used. Variables with some evidence of association (p < 0.10) in these analyses were included in a multiple logistic regression model to identify factors independently associated with choice of health care facility during illness. Odds ratios and adjusted odds ratios are reported with 95% confidence intervals; p-values are based on Wald tests. SPSS (version 12) statistical software was used for all analyses.

Patients were assured that participation would not affect their future care at the clinic in any way. The study was approved by the College of Medicine Research and Ethics Committee and informed consent was received from all participants.

## Results

### Characteristics of the study population

We enrolled 346 patients in the study. The mean age was 39.8 years (range 18-70), 54.3% were female. The mean number of school years completed was 7.8 years. 7.8% had never attended any school, 49.4% only primary school. Travel time to the clinic was longer than 1 hour for 21.4% of the patients, 79.8% were living in urban areas, and 81.5% had been on ART for more than 12 months.

### Health care utilization

Out of the 346 patients, 202 (58%) reported to have had at least one illness in the past 6 months for which they sought medical care. There was no significant difference in frequency of reporting illness episodes between females and males (55.3 vs. 62.0%; p = 0.21). The mean age of those with a reported illness episode was similar to those who did not (40.3 vs. 39.2 years; p = 0.34). Duration on ART, education level, rural/urban residence and length of travel time to the clinic were not significantly associated with reporting an illness episode (statistics not shown). However those who reported an illness episode had a significantly higher socio-economic score than those who did not (5.6 vs. 5.1; p = 0.03). Concerning the choice of health care facility utilized for the reported illness, 117 (57.9%; 95%-confidence interval: 52.7-63.1%) patients said they utilized QECH, while 85 (42.1%; 95%-confidence interval: 36.9-47.3%) went to other facilities, which were: other government hospitals and health centers (n = 43), private hospitals (n = 21), private pharmacies (n = 11), and traditional healers (n = 9). The reasons that ART patients gave for the choice of health care facility during the most recent illness episode are shown in table 1. In univariable analyses, lower education level, rural residence, perceived mild illness of the disease episode, and dissatisfaction with the ART service were associated with utilization of health care facilities other than the own ART clinic, and in the multivariate analysis these factors remained significant (table 2).

**Table 1:** Reasons ART patients stated for utilizing QECH for last illness episode

Reasons for utilizing own clinic^1^	N	%^2^	Reasons for not utilizing own clinic^1^	N	%^3^
Good quality of care	88	75.2	Poor quality of care	13	15.3
Short distance	22	18.8	Long distance	56	65.9
Good attitude of staff	5	4.3	Poor attitude of staff	1	1.2
Referral	4	3.4	Long waiting time	13	15.3
Cost	2	1.7	Cost	1	1.2
Acceptable waiting time	1	0.9	Privacy	0	0
Privacy	1	0.9	Other	25	29.4

1. Patients could give more than 1 reason. 2. Denominator is number of patients who utilized QECH during last illness episode (n = 117). 3. Denominator is number of patients who did not utilize QECH during last illness episode (n = 85).

**Table 2:** Factors associated with utilizing health care facilities other than the own clinic during illness

Factor	HCU^1 ^of QECH^2^	HCU elsewhere	OR^3 ^(95%-CI)	p-value	aOR^4 ^(95%-CI)	p-value
Female gender Male gender	58 59	46 39	1.20 (0.69-2.10)	0.52		
Mean age	40.3	40.2	0.99 (0.97-1.03)	0.97		
Mean education level (years schooling completed)	8.45	7.26	0.93 (0.87-0.99)	0.046	0.92 (0.85-0.99)	0.022
Mean socio-economic score	5.73	5.36	0.91 (0.78-1.05)	0.19		
Rural residence Urban residence	14 103	23 62	2.73 (1.31-5.69)	0.007	2.65 (1.23-5.73)	0.013
Travel time to clinic < 2 hours Travel time to clinic- ≥ 2 hours	109 8	73 12	2.24 (0.87-5.75)	0.094		
Duration on ART < 1 year Duration on ART ≥ 1 year	23 94	15 70	0.88 (0.43-1.80)	0.72		
Perceived severity of illness - mild Perceived severity of illness - severe	85 32	71 13	2.06 (1.00-4.21)	0.049	2.36 (1.09-5.09)	0.029
Satisfied with services of own ART clinic Not satisfied with services of own ART clinic	104 67	13 18	2.15 (0.99-4.67)	0.053	3.41 (1.45-8.01)	0.005

1. HCU, health care utilization. 2. QECH, Queen Elizabeth Central Hospital. 3. OR, odds ratio. 4. aOR, adjusted odds ratio

### Knowledge of service package and perception of the quality of care

Only 6.1% of the 346 patients were aware of all the services available at the ART clinic (treatment of opportunistic infections, malignancies and management of toxicities of ART), while 24.6% knew of none. If aware of any service, most (91.6%) patients said they had this information from the clinic itself, 5.7% from various media and 7.3% from friends. 290 (83.6%) patients reported that they were satisfied with the services provided by the ART clinic. The reasons that patients mentioned for being satisfied or not with the services at the ART clinic are summarized in table 3.

**Table 3:** Reasons for being satisfied or not with service at ART clinic

Reason for being satisfied^1^	N	%^2^	Reason for being dissatisfied^1^	N	%^3^
Improvement after treatment	192	66.2	Long waiting time	40	71.4
Drug availability	180	62.1	Bad attitude of staff	11	19.6
Good attitude of staff	130	44.8	Health workers don't listen to concerns	7	12.5
Timely service	75	25.9	No thorough examination	3	5.4
Health workers listen to concerns	72	24.8	No explanation of diagnoses	3	5.4
Examined thoroughly	27	9.3	No improvement	2	3.6
Clean environment	11	3.8	Inadequate opening hours	1	1.8
Explanation of diagnosis	7	2.4	No access to specialist	0	0
Access to specialist	7	2.4	Unclean environment	0	0
Adequate opening hours	4	1.4	Drug unavailability	0	0

1. Patients could give more than 1 reason. 2. Denominator is number of patients who were satisfied with service (n = 290)

3. Denominator is number of patients who were not satisfied with service (n = 56)

## Discussion

Our results show that patients on longer-term ART frequently seek medical care for intercurrent illnesses. More than 40% of ART patients did not utilize their own clinic during their most recent significant illness episode. Instead they went to other government health centers or hospitals, followed by private hospitals and pharmacies. Long distance to the clinic and to a lesser degree, poor quality of care and long waiting times were the main subjective reasons given, while lower education level, rural residence, perceived mild illness and dissatisfaction with the services of the own ART clinic were independently associated with using health care facilities other than the own clinic. Patients received advice during pre-ART counseling sessions to utilize other clinics for minor illnesses if the ART clinic is far from home and this may have been an important reason why patients did not utilize their own clinic. However our data show that other reasons also play an important role: 37.6% of patients with urban residence and 28.3% with perceived severe illness utilized another clinic, and two factors other than distance to clinic and illness severity were independently associated with using other clinics.

Evaluation of the patterns of health care utilization may give important feed back to ART programs about restrictions in their services and can suggest measures to improve the quality of care. On the basis of our findings we recommend that counseling at the start of ART should stress the importance of consulting one's own ART clinic in case of illness and that if this is not possible, that patients should communicate their ART information to other health care providers. Advising patients to utilize other clinics closer to their residence for minor illnesses carries some risks, such as patients' failure to determine the seriousness on an illness and other clinicians' failure to establish the relevance of an illness in relation to ART toxicity or ART failure. The fact that so many ART patients use various other clinics for intercurrent illnesses underlines the importance of adequate reporting of ART information in health passports, booklets that government health institutions use for documentation of medical events [12]. Furthermore, our findings support the ongoing process of decentralization of ART care, since patients struggle to attend ART clinics that are far from home. Since many clinicians outside recognized ART clinics are confronted with patients on ART it is important that these clinicians will be targeted in ART update training programmes. It is of interest that despite common usage of other health care facilities, satisfaction with clinic services in QECH appears to be high; however few ART patients were aware of the full service package that the ART clinic can offer and therefore these services should be more widely advertised.

There are several limitations to our study. First, the sampling method we used may have led to selection bias, as patients who managed coming to an ART session early may have had different characteristics compared to those who came later. For instance they may have had better access to the facility because of living closer by. Because this sampling method may also favor inclusion of patients who are more likely to utilize QECH during illness, the likely direction of resulting bias would be an underestimation of our main finding, namely that ART patients often utilize other health care facilities than their own clinic. It is however possible that we overestimated the degree of satisfaction with the ART services for the same reason. Secondly, the fact that those who reported illness episodes had a higher mean socio-economic score may indicate that we may not have included illness episodes from those who simply could not afford accessing a health care facility. Alternatively, we did not establish the nature of the illness for which medical care was sought and cannot rule out that events were caused by conditions associated with higher living standards, such as diabetes mellitus or hypertension and their complications. We did not explore whether the other health facilities that patients were utilizing were also ART sites and whether patients had disclosed that they were on ART at the other site. We deliberately analyzed only the last illness because patients would be best able to remember details of the most recent episode. To some degree our results may be specific to the ART clinic of QECH, but in our experience these findings can be extrapolated to urban ART clinics in Malawi and we believe that there are many ART clinics in sub-Saharan Africa in comparable circumstances. Lastly, the cross sectional design of this study limits conclusions on causality and the ability to exclude confounding factors.

## Conclusion

Adult patients on long-term ART in Blantyre, Malawi, frequently seek medical care for intercurrent illnesses in which case many do not utilize services of their own clinic, a phenomenon that was independently associated with rural residence, low education level, perceived mild illness and dissatisfaction with the ART service. This has consequences for ART counseling, reporting of ART information in health passports, and decentralization of ART care.

## Competing interests

The authors declare that they have no competing interests.

## Authors' contributions

All authors contributed to study design, data collection, analysis and interpretation and all were involved in drafting and revising the manuscript; all have given final approval of the version to be published.

## Pre-publication history

The pre-publication history for this paper can be accessed here:

http://www.biomedcentral.com/1472-6963/11/345/prepub
